# Supplementary material for: Chemical Profile and Biological Activities of Brassica rapa and Brassica napus Ex Situ Collection from Portugal
Source: Foods. 2024 Apr 11;13(8):1164. doi: 10.3390/foods13081164 (PMC11049378; doi:10.3390/foods13081164)
Supplement: Supplementary file 1 [file foods-13-01164-s001.zip › foods-2931600-supplementary.pdf]

foods

Supplementary Materials

Chemical Profile and Biological Activities of *Brassica rapa* and *Brassica napus* Ex Situ Collection from Portugal

Carmo Serrano [1,2,*], M. Conceição Oliveira [3], V. Rolim Lopes [6], Andreia Soares [1], Adriana Molina[4,5], Beatriz H. Paschoalinotto[4,5], Tânia C.S.P.Pires [4,5], Octávio Serra[6] and Ana M. Barata [6]

1 Instituto Nacional de Investigação Agrária e Veterinária (INIAV, I.P.), Av. da República, 2780-157 Oeiras, Portugal; margarida.sapata@iniav.pt (M.S.); andreia.soares@iniav.pt (A.S.)
2 LEAF–Linking Landscape: Environment, Agriculture and Food–Research Center, Instituto Superior de Agronomia, Associated Laboratory TERRA, Universidade de Lisboa, Tapada da Ajuda, 1349-017 Lisboa, Portugal
3 Centro de Química Estrutural, Institute of Molecular Sciences, Instituto Superior Técnico, Universidade de Lisboa, 1049-001 Lisboa, Portugal; conceicao.oliveira@tecnico.ulisboa.pt (M.C. Oliveira)
4 Centro de Investigação de Montanha (CIMO), Instituto Politécnico de Bragança, Campus de Santa Apolónia, 5300-253 Bragança, Portugal; carlap@ipb.pt (C.P.); ruiabreu@ipb.pt (R.M.V.A.); lillian@ipb.pt (L.B.)
5 Laboratório Associado para a Sustentabilidade e Tecnologia em Regiões de Montanha (SusTEC), Instituto Politécnico de Bragança, Campus de Santa Apolónia, 5300-253 Bragança, Portugal
6 Banco Português de Germoplasma Vegetal (BPGV), INIAV I.P., Qta S. José, S. Pedro Merelim, 4700-859 Braga, Portugal
* Correspondence: carmo.serrano@iniav.pt

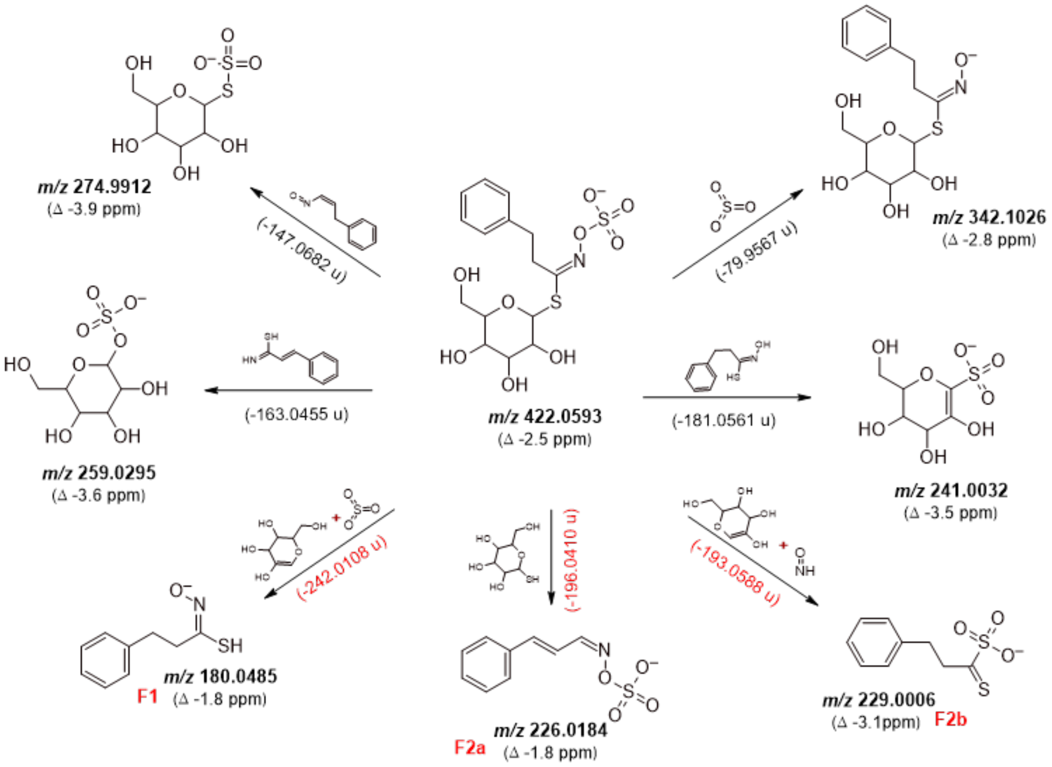

Scheme S1 – Proposed fragmentation mechanism for precursor ion *m/z* 422.0593 attributed to the deprotonated molecule of Gluconasturtiin
